# HIV-1 and HTLV-1 Transmission Modes: Mechanisms and Importance for Virus Spread

**DOI:** 10.3390/v14010152

**Published:** 2022-01-14

**Authors:** Svetlana Kalinichenko, Dmitriy Komkov, Dmitriy Mazurov

**Affiliations:** 1 Cell and Gene Technology Group, Institute of Gene Biology RAS, 119334 Moscow, Russia; skalin13@gmail.com; 2 Center for Precision Genome Editing and Genetic Technologies for Biomedicine, Institute of Gene Biology RAS, 119334 Moscow, Russia; dmitkomserg@gmail.com

**Keywords:** HIV-1, HTLV-1, cell-to-cell transmission, virological synapse, filopodia, cytonemes, replication-dependent vectors

## Abstract

So far, only two retroviruses, human immunodeficiency virus (HIV) (type 1 and 2) and human T-cell lymphotropic virus type 1 (HTLV-1), have been recognized as pathogenic for humans. Both viruses mainly infect CD4+ T lymphocytes. HIV replication induces the apoptosis of CD4 lymphocytes, leading to the development of acquired immunodeficiency syndrome (AIDS). After a long clinical latency period, HTLV-1 can transform lymphocytes, with subsequent uncontrolled proliferation and the manifestation of a disease called adult T-cell leukemia (ATLL). Certain infected patients develop neurological autoimmune disorder called HTLV-1-associated myelopathy, also known as tropical spastic paraparesis (HAM/TSP). Both viruses are transmitted between individuals via blood transfusion, tissue/organ transplantation, breastfeeding, and sexual intercourse. Within the host, these viruses can spread utilizing either cell-free or cell-to-cell modes of transmission. In this review, we discuss the mechanisms and importance of each mode of transmission for the biology of HIV-1 and HTLV-1.

## 1. Brief Comparative Biology of HIV-1 and HTLV-1

### 1.1. Entry

**Figure 1:**
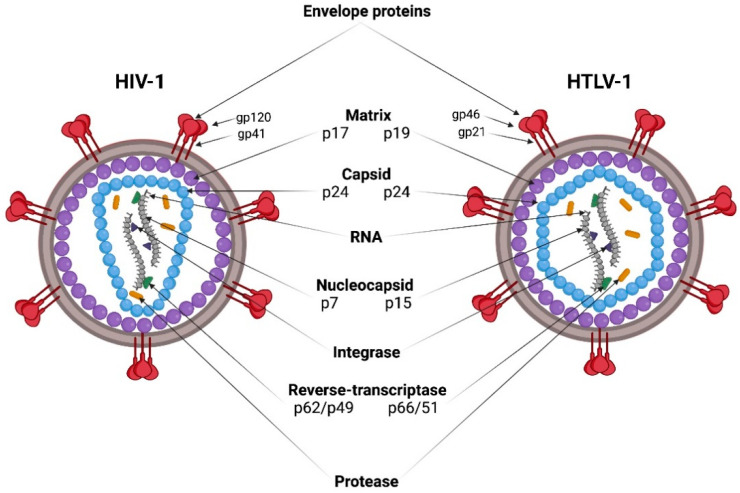
HIV-1 and HTLV-1 virion composition. Subunit names of structural and nonstructural proteins are given for each virus. Created with BioRender (https://biorender.com/) on 26 November 2021.

HIV-1 and HTLV-1 viral particles have a spherical shape with a diameter of 80–100 nm, and are covered with a bilayer lipid membrane originating from the plasma membrane of an infected cell during the assembly and release of virions (Figure 1). The lipid bilayer contains envelope glycoprotein (Env) complexes. Env is a trimer of heterodimers, consisting of two non-covalently associated subunits: surface (SU) and transmembrane (TM). A layer of matrix protein (MA) lies under the virion membrane, and surrounds the capsid core. The capsid core is formed by the capsid protein (CA), which, after virion maturation, condenses into a truncated cone (HIV-1) or icosahedron (HTLV-1). The capsid core contains two copies of positive single strand genomic RNA (gRNA) in complex with the nucleocapsid protein (NC) and replication enzymes reverse transcriptase (RT) and integrase (IN) [1,2] (Figure 1).

Like many enveloped viruses, mature HIV-1 and HTLV-1 virions bind and fuse with the target cell membrane through the interaction of Env with cellular receptors. The binding of the SU subunit to the receptors causes conformational changes in Env, resulting in TM-mediated fusion of the viral and cellular membranes. The molecular mechanism of HIV-1 entry into the cell has been described in detail [3]. The binding of HIV-1 Env SU (gp120) to the T-cell receptor CD4 results in conformational changes in Env, and the recruitment of the coreceptors C-C chemokine receptor type 5 (CCR5) or C-X-C chemokine receptor type 4 (CXCR4). The type of coreceptor usage determines the tropism of the virus; therefore, strains of HIV-1 may be CCR5-tropic, CXCR4-tropic, or R5X4 dual-tropic. After gp120 binds to the coreceptor, HIV-1 Env TM (gp41) induces fusion. A capsid containing two copies of gRNA, RT, and IN enters the cytoplasm [4]. According to the latest observations, the intact or almost intact HIV-1 capsid is translocated into the nucleus, where the reverse transcription of gRNA is completed (reviewed in [5]). It remains unclear where HTLV-1 capsid core uncoating occurs. After viral gRNA reverse transcription, proviral DNA integrates into the transcriptionally active sites within the host chromatin.

HTLV-1 Env interacts with three receptors: heparan sulfate proteoglycan (HSPG), neuropilin 1 (NRP-1), and glucose transporter type 1 (GLUT1) [6]. HTLV-1 Env SU (gp46) initially binds to HSPG, leading to subsequent NRP-1 recruitment. In a complex with HSPG/NRP-1, gp46 is assumed to change its conformation, and facilitate GLUT1 binding to TM (gp21). This activates the fusogenic peptide of gp21, ultimately leading to the fusion of viral and cellular membranes [7].

### 1.2. Genomes

**Figure 2:**
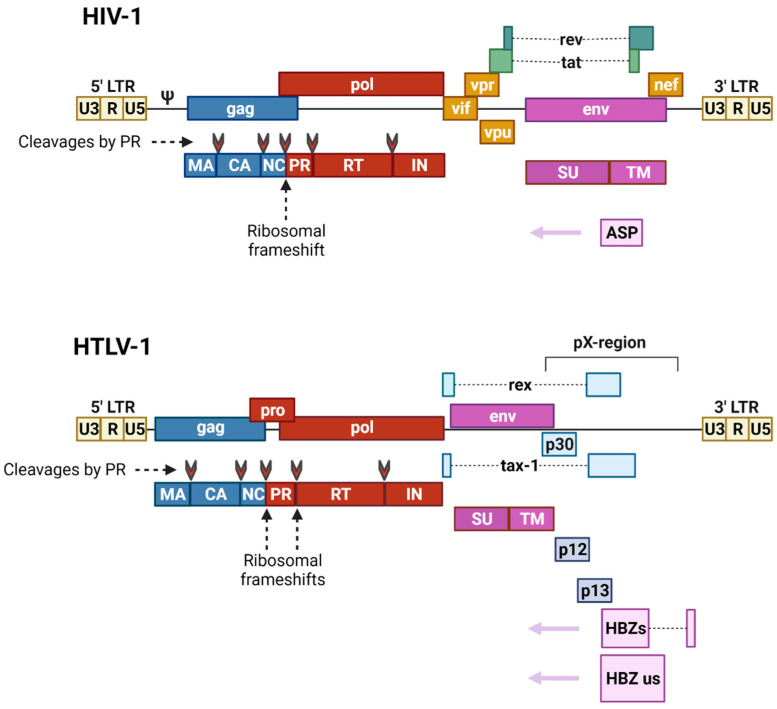
Comparison of genome organization for HIV-1 (top) and HTLV-1 (bottom). The names of viral genes and proteins are given in lowercase and uppercase letters, respectively. The arrowheads indicate protein cleavage sites. This image was created using BioRender (https://biorender.com/) on 26 November 2021.

The genomes of HIV-1 (9.75 kb) and HTLV-1 (8.5 kb) contain the main retroviral genes *gag*, *pro*, *pol*, and *env*, and are flanked by long terminal repeats (LTRs) of about 600 nt [8,9]. LTRs facilitate viral integration into the host genome, and contain promoter elements, polyadenylation signals, and other regulatory sequences necessary for viral RNA transcription. In addition to the structural proteins (group-specific antigen (Gag) and Env), and enzymatic proteins (protease (Pro) and polymerase (Pol)), the gRNA of HIV-1 encodes the regulatory proteins (transcriptional transactivator (Tat) and regulator of expression of viral proteins (Rev)), as well as the accessory proteins (negative effector (Nef), viral infectivity factor (Vif), viral protein r (Vpr), and viral protein u (Vpu)). Tat activates transcription from the LTRs, and Rev modulates the transport of viral mRNA from the nucleus to the cytoplasm. Nef, Vif, Vpr, and Vpu are not involved in the expression of viral genes, but play an important role in the pathogenesis of AIDS, and the evasion of the host’s immune response [9]. The minus DNA strand of HIV-1 contains the ORF located at the gp120/gp41 junction of the *env* gene, and encodes a highly hydrophobic antisense protein (ASP) with no known homologs. ASP has been found in the nucleus, on the surface of infected cells, as well as on virions, and its function is largely unknown [10] (Figure 2 top).

Regulatory Tax, Rex and accessory/auxiliary p12, and p13 and p30 HTLV-1 proteins are encoded by sequences located in the gRNA in different reading frames between *env* and the 3′-LTR (pX region). Tax is analogous to HIV-1 Tat, and Rex is analogous to HIV-1 Rev. The HTLV-1 provirus also transcribes the minus-strand RNA, which encodes the HTLV-1 basic leucine zipper factor protein Hbz and its splicing isoform, HBz-SI [11] (Figure 2 bottom).

### 1.3. Assembly of Viral Particles

The proviruses of HIV-1 and HTLV-1 are transcribed into gRNA and unspliced and spliced mRNA. The surface glycoprotein, Env, is synthesized in the endoplasmic reticulum as the precursor polyprotein, gp160 (HIV-1) or gp66 (HTLV-1), and delivered to the cell membrane by the Golgi apparatus. During this translocation, Env is cleaved into gp120 and gp41 (HIV-1) or gp46 and gp21 (HTLV-1) by the host furin protease. Trimeric Env complexes are incorporated into the outer surface of the plasma membrane at the viral particle assembly sites.

During HIV-1 replication, the precursor polyproteins, Gag and GagPol, are translated in a ratio of ~20:1 from the same mRNA, with the GagPol polyprotein being produced as a result of a minus one nucleotide shift of the reading frame during Gag translation [12]. The Gag polyprotein contains the MA, CA, NC, and p6 domains, whereas the GagPol polyprotein contains the MA, CA, NC, PR, RT, and IN domains. These proteins are translocated to the inner surface of the plasma membrane, and accumulate in lipid rafts [13], where they are assembled into a hexameric lattice that induces a curvature of the plasma membrane. The binding of Gag to the membrane is mediated by the interaction of the MA domain with phosphatidylinositol 4,5-biphosphate (PI(4,5)P2) [14]. The CA domain facilitates the multimerization of Gag, and the NC domain recruits dimeric viral gRNA into the forming virions.

**Figure 3:**
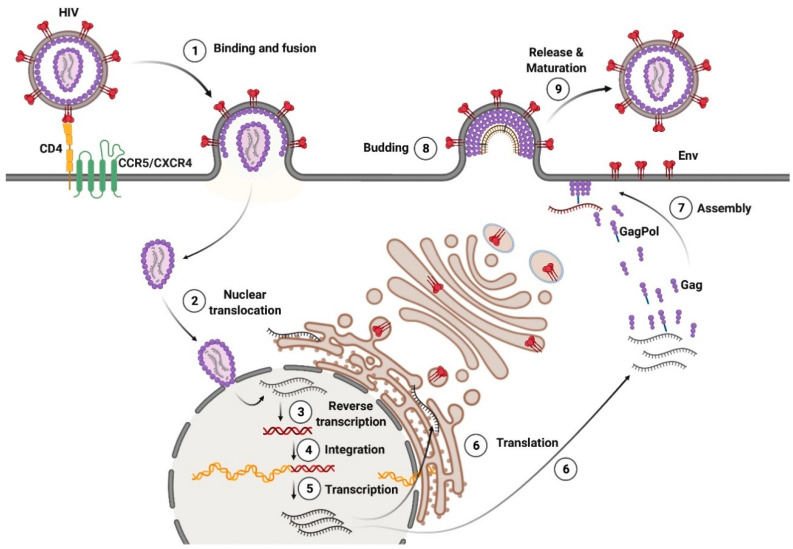
HIV-1 viral life cycle, from entry to production of mature virions. See explanations in the text. Adapted from “HIV Sites for Therapeutic Intervention” by BioRender.com (2021). Retrieved from https://app.biorender.com/biorender-templates, accessed on 26 November 2021.

The p6 domain of the Gag polyprotein recruits endosomal sorting complexes required for transport (ESCRT) to the assembly site, which catalyzes membrane scission during the budding of viral particles. Immature HIV-1 viral particles are released from the cell surface, and undergo proteolytic maturation. The maturation of the viral particles is mediated by the viral protease, PR, which is a part of the Pol precursor protein together with RT and IN, and is autocatalytically excised from the GagPol polyprotein. Gag is cleaved by PR into the proteins, MA, CA, NC, and p6, whereas GagPol is cleaved into MA, CA, NC, RT, and IN. The processing of Gag and GagPol induces the disassembly of the membrane-bound Gag lattice, and the assembly of the mature conical core from the condensed capsid protein CA. The capsid packs two single-stranded copies of viral gRNA along with NC, RT, and IN (reviewed in [15]) (Figure 3).

Though the *pro* and *pol* genes are located in the same reading frame in the HIV-1 genome, the viral protease is encoded by a separate gene in the HTLV-1 genome. During HTLV-1 provirus translation, the GagPro transcript is created by a minus one nucleotide frameshift, and the GagProPol transcript is generated by a minus two nucleotides frameshift [12]. HTLV-1 Gag accumulates and multimerizes on the inner surface of the plasma membrane, not in lipid rafts like HIV-1 Gag, but in tetraspanin microdomains as a result of the interaction of the MA domain with the intracellular loop of the tetraspanins, CD81 and CD82 [16,17]. The released virions undergo proteolytic maturation into mature infectious particles as a result of the cleavage of the Gag, GagPro, and GagProPol proteins by the viral protease and the rearrangement of the capsid protein [18].

## 2. Modes of HIV-1 and HTLV-1 Transmission

Viruses can spread in two ways: as free viral particles or by direct cell-to-cell transfer. Free virions must face innate and acquired immune defenses, diffusion in the environment, and the need to overcome long distances to reach target cells. Cell-to-cell transmission may enhance the multiplicity of infection, and allows host immune response evasion, as well as more rapid viral replication kinetics [19]. HIV-1 uses both modes of transmission to efficiently replicate in vitro and *in vivo*. HTLV-1 is transmitted mainly through cell-cell contacts. The exceptions are dendritic cells (DCs), which can get infected with free virions. HTLV-1-infected T cells have been shown to produce very few free virions in vitro, of which only 1 out of 10^5^–10^6^ is infectious [20,21]. In the plasma of HTLV-1 infected subjects, the viral RNA is rarely detected [22]. Therefore, in contrast to HIV-1, cell-free blood products carry minimal risk of HTLV-1 infection. Within individuals, HTLV-1 is also spread by the clonal expansion of infected T cells.

## 3. Methods for Cell-to-Cell Infectious Transmission Measurement

Various approaches have been developed for the quantitative assessment of cell-to-cell transmission using experimental and mathematical analyses, fluorescent substrates or labels, and reporter systems. Dimitrov et al. investigated the kinetics of the accumulation of HIV-1 viral particles in cell culture supernatant during several rounds of infection. As a result, the equation *k* = ln(*n*)/*t_i_* was derived, where *k* is the rate constant of infection, *n* is the number of infectious particles produced by one cell, and *t_i_* is the duration of one cycle of viral replication. Using this equation, the infectivity of virus-producing cells was calculated as being 100–1000 times higher than the infectivity of free viral particles produced by the same cells [23].

To discriminate between cell-free and cell-to-cell modes of dissemination, Sourisseau et al. established an assay in which the cells are cultured under a shaking condition [24]. The authors supposed that gentle shaking blocks cellular contact formation and cell-to-cell transmission. HIV-1-infected cells were mixed with fluorescent dye-labeled recipient T cells, and the kinetics of viral replication in static and when shaking lymphocyte cultures was measured by flow cytometry. The obtained results demonstrated the predominant transmission of HIV-1 via cell contacts. Similarly, gentle shaking to inhibit cell contact formation has been utilized for the quantitative evaluation of viral transmission using experimental–mathematical modeling [25]. Monitoring of the dynamics of virus spread in Jurkat cells infected with HIV-1, and cultivated under static or shaking conditions, revealed that cell-to-cell transmission causes 60% of viral infection, shortens the generation time of the virus 1.1-fold, and increases the viral fitness 3.9-fold. However, Chazal et al. reported later that the poor efficiency of HIV-1 replication in the shaking culture of HIV-1-infected T cells was related to the loss of infectivity of cell-free virions produced by shaky lymphocytes [26]. Hence, the results from shaking experiments should be interpreted cautiously.

Cavrois et al. developed a β-lactamase assay to evaluate HIV-1 transmission. The detection system consists of the chimeric protein, β-lactamase-Vpr (BlaM-Vpr), which is readily incorporated into HIV-1 virions, and the vital fluorescent substrate, CCF2/AM, added to the recipient cells. When CCF2/AM enters the cytoplasm of target cells, BlaM cleaves the β-lactam ring of CCF2, which causes a change in the fluorescence emission spectrum from green (520 nm) to blue (447 nm). BlaM analysis allows the detection and quantification of the transmission of viral particles by fluorescence microscopy, flow cytometry, or UV photometry [27]. Variations of BlaM analysis and examples of its use are described in detail in the review by Jones and Padilla-Parra [28].

To track HIV replication in live cells, Chen et al. developed the HIV-1 Gag-iGFP molecular clone, in which green fluorescent protein (GFP) was embedded between the MA and CA of Gag. Each viral particle contained ~5000 GFP molecules, making it possible to visualize the virus with a high degree of sensitivity, including the transfer of HIV-1 into recipient T cells labeled with the red fluorophore, chloromethyl benzoyl aminotetramethyl rhodamine (CMTMR) [29]. Measurement of the viral fluorescence in target cells revealed that cell-to-cell transmission is 18,000 times more efficient than infection with free virions.

To assess cell infection with free virus, and to study the early stages of viral replication, Derse et al. developed a sensitive quantitative virus-like particle (VLP) assay using HTLV-1-based vectors [20]. The VLPs were obtained by transfecting cells with three plasmids: a packaging plasmid encoding structural and regulatory HTLV-1 proteins, a reporter plasmid encoding firefly luciferase (Luc) fused in frame with yellow fluorescent protein (YFP), and an Env expression plasmid. The target cells were incubated with supernatant containing VLPs. The efficiency of infection was quantified based on the activity of luciferase in the cell extract, or by counting the number of infected cells using flow cytometry. This analysis showed that the infectivity of purified HTLV-1 VLPs is 1000 times lower than the infectivity of isolated HIV-1 VLPs.

However, such a reporter system is not suitable for the quantitative assessment of HIV-1 and HTLV-1 cell-to-cell transmission, since the reporter signal is detected not only in infected target cells, but also in transfected producer cells. To solve this problem, we previously developed the intron-regulated reporter vectors, HTLV-1 inLuc and HIV-1 inLuc, as well as HTLV-1 inYFP and HIV-1 inYFP. When these vectors were used together with the packaging and Env-encoding plasmids, the reporter signal was only detected in target cells after one cycle of viral replication was completed [30]. This was achieved by adapting a concept developed for the detection of retrotransposition events [31]. Specifically, the reporter gene was inactivated in transfected cells by using two approaches: first, the reporter expression cassette was reversed relative to the viral genomic sequence, and second, the reporter gene was interrupted by inserting an intron of beta-globin. Importantly, the intron was placed in the same orientation as the viral genome, thereby allowing its removal from U3-driven reporter RNA followed by the packaging of spliced RNA into VLPs. Thus, neither CMV- nor U3-driven mRNA was capable of translating active reporter protein in cells producing VLPs. During the infection of a target cell, the spliced reporter RNA is reverse transcribed, and now encodes a functional reporter that can be detected after the integration of the transfer vector into the host genome and its subsequent expression. This design, therefore, allows reporter activation only in target cells, and enables the detection of infection events in co-cultures of VLP-producing (transfected) cells with permissive target cells with a high degree of accuracy. Using this system, it has been shown that the efficiency of cell-to-cell infection of HIV-1 is twofold higher, and that of HTLV-1 is 10,000-fold higher, in comparison to infection mediated by cell-free viral particles. Moreover, in contrast to HIV-1, a strong dependence of HTLV-1 transmission on the formation of cell-cell contacts, and the expression of the viral Tax protein was demonstrated. Later, the intron-regulated vectors were improved by optimizing the splicing efficiency of the reporter RNA packaged into viral particles [32]. Recently, we pseudotyped HIV-1 particles with SARS-CoV-2 protein S, and, using the HIV-1 replication-dependent reporter vector, inLuc, demonstrated that the cell-to-cell infection of SARS-CoV-2 was about one order of magnitude less sensitive to neutralization by convalescent sera than cell-free infection. The observed differences were even more pronounced when infection tests were performed using replication-competent coronavirus [33]. This indicates that newly emerging viruses can also adopt mechanisms of cell-to-cell transmission to enhance their spread, and escape from immune control.

## 4. Mechanisms of Intercellular Retrovirus Transmission

Below, we review the types of intercellular contacts that have been observed and described as structures capable of transmitting HIV-1 and HTLV-1 virions from donor cells to recipient cells.

### 4.1. Virological Synapse (VS)

**Figure 4:**
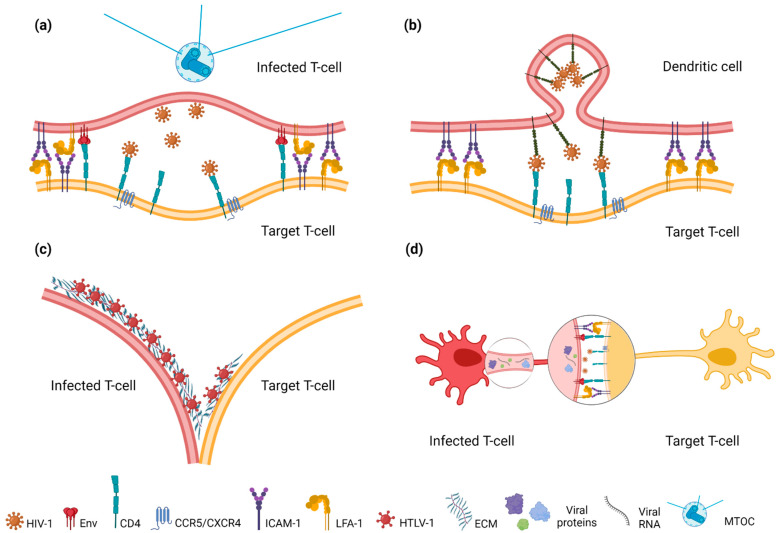
A schematic demonstration of transmission-capable intercellular contacts forming (**a**) virological synapse (VS), (**b**) infectious synapse (IS), (**c**) viral biofilms, and (**d**) conduit-based mini-VS. Created with BioRender (https://biorender.com/) on 20 December 2021.

The VS is a cytoskeleton-dependent, stable adhesive junction, across which a virus is transmitted by direct transfer [4] (Figure 4a). The VS received its name due to its similarity to an immunological synapse, the formation of which is induced by the binding of the major histocompatibility complex (MHC) on the surface of antigen-presenting cells (APCs) to the T-cell receptor (TCR) on the surface of CD3 lymphocytes. The VS was first discovered in 2003 in Charles Bangham’s laboratory through confocal microscopy of T lymphocytes obtained from HTLV-1-infected patients co-cultured with T lymphocytes isolated from healthy donors [34]. One year later, Jolly et al. described a VS between HIV-1-infected Jurkat T cells and primary CD4+ T cells [35].

VS formation is initiated by the interaction of Env with the corresponding cellular receptor, and is stabilized by the binding of intercellular adhesion molecule 1 (ICAM-1) to lymphocyte function-associated antigen 1 (LFA-1) [36]. The binding of ICAM-1 to LFA-1 leads to the polarization of the microtubule-organizing center (MTOC) in the infected (donor) cell towards the recipient (target) cell [37,38]. MTOC polarization facilitates the actin- and tubulin-dependent accumulation of Gag, Env, and viral RNA in the intercellular contact zone [34,39]. Virus transfer across the synaptic cleft occurs within 1–3 h, after which Gag is detected in the target cell [35,40].

In HTLV-1-infected T cells, MTOC polarization is triggered by the activation of the ICAM-1-mediated Ras-MEK-ERK signaling pathway [41]. The viral accessory protein, Tax, acts synergistically with ICAM-1, activating the CREB signaling pathway. This activation increases the expression of the GTP-binding protein Gem, which regulates cytoskeleton reorganization and cell migration [42]. Tax also induces the expression of actin-bundling protein fascin [43], and secretion of leukotriene B4 [44], which have been implicated in HTLV-1 transmission. In contrast to HTLV-1, MTOC polarization in HIV-1-infected T cells is triggered via the LFA-1 molecule, and the activation of a downstream T cell-specific ZAP70 kinase signaling cascade [38]. Since both LFA-1 and ICAM-1 are expressed on T cells, it can be hypothesized that the cell-to-cell transmission of the virus in T lymphocytes is triggered by both ICAM-1- and LFA-1-mediated signaling pathways. However, this should be confirmed experimentally.

It has also been reported that HIV-1-infected T cells can form multiple synapses [45]. In this case, the Gag protein accumulated in ring-shaped structures at sites of intercellular contact without MTOC polarization.

The binding of virion-associated HIV-1 Env to CD4 triggers fusion. However, during cell-to-cell transmission, this process is inhibited by cell membrane proteins that accumulate at the VS zone: tetraspanins [46], ezrin [47], and Ewi-2 [48]. In addition, the fusogenic activity of Env is inhibited by the fixation of the cytoplasmic domain of gp41 in the multimeric lattice of Gag. Moreover, the fusogenic capacity of Env is activated during the proteolytic maturation of viral particles [49,50]. All these mechanisms prevent syncytia formation during VS establishment.

Two mechanisms of HIV-1 transmission across the VS have been described: endocytosis, and fusion with the plasma membrane. According to the model of Chen et al. [29], HIV-1 viral particles are internalized from the synaptic cleft into the early endosomes of the target cell, where they undergo proteolytic maturation followed by fusion with the endosomal membrane [49,51,52]. However, other groups failed to detect HIV-1 endocytosis at the VS site by confocal or electron microscopy with tomography, indicating that the virus enters target cells by fusion with the plasma membrane [35,53]. Puigdomenech et al. hypothesized that the mechanism of virus entry depends on cell type. The virus enters primary CD4+ T cells mainly via endocytosis and lymphoblasts, or T cell lines via fusion with the plasma membrane [54]. Quentin Sattentau hypothesized that the membrane entry site at the synaptic cleft depends on the cultivation conditions [55]. Chronically infected CD4+ T cells produce a large amount of mature viral particles. In this case, after the formation of the VS, mature virions fuse with the cell membrane. If the VS forms during acute infection of primary cells, immature and fusogenically incompetent virions are released into the synaptic cleft. Such viral particles are internalized into early endosomes, where they mature, fuse with the endosomal membrane, and are released into the cytoplasm.

### 4.2. Infectious Synapse (IS)

The IS is an Env/CD4-independent type of VS that mediates HIV-1 and HTLV-1 transmission from APCs to T cells (Figure 4b). APCs, such as DCs and macrophages, are the first targets of HIV-1 and HTLV-1 during transmission via sexual routes and breastfeeding [56,57,58,59]. It has been reported that CD4-negative cells of the mucous epithelium express the glycosphingolipid galactosylceramide (GalCer), which interacts with HIV-1 Env [60]. After binding to GalCer, and crossing the epithelial barrier by transcytosis, R5-tropic HIV-1 is captured by subepithelial DCs and macrophages in a CD4-independent manner via alternative receptors. The DCs then migrate to the lymph nodes, where they infect T cells [61].

It should be noted that viral replication is inhibited in mature DCs and macrophages by restriction factors [62]. For example, sterile alpha motif and HD domain 1 (SAMHD1) inhibits reverse transcription by depleting the cellular pool of deoxynucleotide triphosphates [63,64], and the cytidine deaminase, APOBEC3G, induces hypermutation of the viral genome [65]. Therefore, the mode of HIV-1 transmission depends on DC maturation status. In *trans*-infection, immature and mature DCs transmit captured HIV-1 to T cells within the first 24 h without being infected themselves [66,67,68]. Later, immature DCs become productively infected, and transmit viral particles produced de novo to T cells in the *cis*-infection process [66,67,69,70]. The healthy mucosa contains immature DCs, whereas the lymph nodes and gut-associated lymphoid tissue contain mature DCs. Therefore, different mechanisms of HIV-1 transmission are possible at different anatomic sites in vivo [66].

In 2008, Kathryn Jones and colleagues demonstrated for the first time that purified HTLV-1 virions, though having low infectivity for T lymphocytes, efficiently infect DCs of monocytic origin (MDDCs), as well as plasmacytoid and immature myeloid DCs [71]. Later, Alais et al. demonstrated that MDDCs were significantly more susceptible to HTLV-1 infection with virus embedded in biofilm (see below) than with cell-free virus. The susceptibility of MDDC to HTLV-1 infection can be explained by a high level of neuropilin-1 expression, which is barely detectable on primary activated lymphocytes [58]. Different DC subsets are not equally susceptible to HTLV-1 infection. Rizkallah et al. used in vitro generated monocyte-derived IL-4 DCs, TGF-β DCs, and IFN-α DCs that mimicked myeloid DCs of the blood, mucosal DCs of the gut, and inflammatory DCs found in injured skin, respectively. Authors have shown that both TGF-β DC and IL-4 DC were susceptible to HTLV-1 infection, whereas IFN-α DCs were resistant [72]

HTLV-1, like HIV-1, crosses the epithelial barrier by transcytosis, and is captured by DCs, which can later become productively infected [73]. As with HIV-1 transmission, there are two phases of HTLV-1 transmission between immature DCs and T cells. *Trans*-infection takes place immediately after the virions are captured, followed later by *cis*-infection by viral particles synthesized de novo [71]. DC-specific ICAM-3-grabbing nonintegrin (DC-SIGN) is a key receptor mediating both processes. It is a C-type mannose-binding lectin that is expressed on the surface of MDDCs, some macrophages, and activated B cells. The inhibition of DC-SIGN significantly attenuates both productive MDDC infection and HTLV-1 transmission between MDDCs and T lymphocytes [74].

DC-SIGN was first discovered as a receptor that interacted with ICAM-3 during the formation of contacts between DCs and T cells [75]. Later, it was reported that DC-SIGN regulates DC migration by binding to ICAM-2, which is expressed on the surface of vascular endothelial cells [76]. DC-SIGN recognizes mannose oligosaccharides on the surface of bacterial and viral pathogens, and mediates the uptake and degradation of the latter in late endosomes/lysosomes, as well as the presentation of the corresponding antigens to T cells. However, some pathogens can avoid lysosomal degradation [77]. DC-SIGN binding to HIV-1 gp120 and HTLV-1 gp46 has been shown to mediate viral particle capture, internalization, and transmission to T cells [74,78,79,80]. However, the results of other studies indicate the existence of a DC-SIGN-independent mechanism of *trans*-infection with HIV-1 viral particles [81,82,83,84].

Another molecule that mediates the uptake and transmission of HIV-1 is the type I lectin receptor CD169/sialic acid-binding immunoglobulin-type lectin 1 (Siglec-1). This receptor is expressed on the surface of activated mature DCs and macrophages, and binds to the GM3 ganglioside in the HIV-1 viral envelope [85,86,87]. In complex with Siglec-1, HIV-1 viral particles accumulate in deep invaginations of the plasma membrane, known as virus-containing compartments (VCCs), where they are protected from lysosomal degradation and neutralizing antibodies [85,88]. Then, HIV-1 viral particles are translocated from the VCC to the IS for transmission.

The IS was first visualized in 2003 by McDonald et al. via fluorescence microscopy of conjugates formed between HIV-1-loaded MDDCs and T cells. Within a few minutes after the formation of the cell conjugates, the accumulation of CD4, CCR5, CXCR4, and HIV-1 was observed at the intercellular contact sites, and the captured HIV-1 particles were located on or near the MDDC surface [89]. Later, Felts et al. analyzed the spatial architecture of the IS and HIV-1 distribution using ion abrasion scanning electron microscopy, electron tomography, and super-resolution light microscopy. Their study revealed membrane extensions derived from DCs that enveloped the T cell, and shielded the synapse zone. Furthermore, filopodial extensions emanating from T cells that contacted virions located deep inside the invaginations of the DC plasma membrane were visualized [90]. The formation of the IS and conduits (discussed below) during bidirectional HTLV-1 transmission between DCs and T cells was observed by Shimauchi et al. using confocal and electron microscopy [91].

The formation of the IS during HIV-1 transmission is Env- and CD4-independent. The adhesion molecules, ICAM-1 and LFA-1, play a key role in this process [92]. However, the interaction of Env with CD4 is necessary for the transfer of HIV-1 viral particles across the synaptic cleft towards the target cell [90]. Three models describing HIV-1 transmission through the IS have been proposed: from endosomes, from the surface of DCs, and from VCCs.

According to the endosomal model, DCs capture and internalize HIV-1 particles in non-lysosomal intracellular compartments where the virions are protected from proteolytic cleavage. After the formation of the IS, viral particles are translocated into the synaptic cleft by exocytosis [67,68,84,93,94]. In a recent work by Bayliss et al., the depletion of some cellular proteins involved in endosomal trafficking prevented the translocation of HIV-1 viral particles from intracellular compartments to the plasma membrane, and significantly reduced *trans*-infection [95].

The model of transmission from the DC surface suggests that the virions involved in *trans*-infection are located on the plasma membrane rather than being delivered from intracellular compartments. This model is based on a report by Cavrois et al. showing that neutralization of the DC surface-bound HIV-1 virions with soluble CD4 completely prevented the *trans*-infection of T cells [66].

The VCC transmission model is based on the results of fluorescence microscopy, which revealed the accumulation of HIV-1 virions in the invaginations of the plasma membrane of lipopolysaccharide-activated and myeloid DCs. Upon the contact of DCs with T cells, VCCs were polarized to the IS, ensuring the delivery of viral particles for transmission [96]. Furthermore, endocytosis of HIV-1 viral particles from the VCC, induced by a modification in the cytoplasmic domain of Siglec-1, significantly decreased the *trans*-infection of T cells [88].

### 4.3. Viral Biofilms

The extracellular matrix (ECM) is a three-dimensional network composed of extracellular molecules of high molecular weight that are located on the surface of bacterial and eukaryotic cells. The composition of the ECM varies depending on the type of cells producing it, but its function is quite fixed, and includes participation in cell adhesion, intercellular communication, and differentiation. Bacterial ECM is a key component of bacterial biofilms which comprise an aggregation of bacteria attached to each other and to a certain surface (reviewed in [97]).

To date, HTLV-1 is the only virus known to form a similar structure on the surface of infected cells (Figure 4c). HTLV-1 biofilm-like structures were initially described by Pais-Coreia et al. [98]. Using confocal and electron microscopy, they detected clusters of viral particles embedded in the ECM on the surface of HTLV-1-infected T cells. The transactivating protein Tax plays a central role in the production of ECM components by infected cells and the formation of HTLV-1 biofilms. Cell-surface HTLV-1 viral clusters are enriched with glycoproteins and virally-induced ECM components, such as collagen, fibronectin, agrin, and sialyl-LewisX (sLeX). The tetrasaccharide, sLeX, mediates the adhesion of lymphocytes. Agrin is the heparan sulfate proteoglycan that links cellular receptors, and is involved in the formation of neuronal, immunological, and virological synapses. Additionally, the viral clusters were found to be enriched with galectin-3, which forms the ECM lattice, and tetherin, which retains budding viral particles on the cell surface [99]. The viral biofilms also include O-glycosylated cellular receptors CD43/sialophorin and CD45/phosphotyrosine phosphatase [100]. Unlike HIV-1 Env, which contains ~25 glycosylation sites [101], and forms a “glycan shield” protecting the virus from neutralizing antibodies [102], HTLV-1 Env contains only five sites for N-glycan attachment [103]. Hence, it is tempting to speculate that embedding the viral particles into the carbohydrate-rich ECM helps HTLV-1 to avoid immune recognition.

The exact mechanism of HTLV-1 transmission using biofilms has not yet been identified. It was reported that viral biofilms localized mainly next to the VS, and that ECM components, together with viral particles, were quickly (within 5 min of cell co-cultivation) transferred to the surface of target cells. Only a small portion of viral particles was transmitted across the VS. Removal of the biofilms by washing with heparin reduced the infectious capacity of HTLV-1-producing cells by 80%, and the residual infectivity correlated with the amount of Gag remaining on the cell surface [98]. It is still unclear whether biofilm-associated HTLV-1 viral particles fuse with the plasma membrane of target cells, or are internalized and enter the cytoplasm after fusion with the endosomal membrane.

Thus, viral biofilms such as the VS and IS promote virion accumulation, and may help protect HTLV-1 virions from immune attack, and enhance the cell-to-cell transmission of HTLV-1. However, the lack of studies in this area during the past few years challenges the importance of viral biofilms for HTLV-1 infection.

### 4.4. Membrane Protrusions (Filopodia and Tunneling Nanotubes (TNT)/Intercellular Conduits) and Exosomes

Evidence that membrane protrusions can serve as an avenue for retrovirus movement was first obtained in Walter Mothes’ laboratory [104]. The authors showed that HEK 293T cells expressing the corresponding viral receptors used filopodia to capture murine leukemia virus (MLV), avian leukemia virus (ALV), or HIV-1 viral particles added to the culture medium. The viruses then “surfed” along the filopodia, and fused with the cell body. Subsequently, these authors observed the formation of filopodia bridges (cytonemes) between retrovirus-infected and uninfected cells [105]. Cytonemes extended from the uninfected cell, and connected to the surface of the infected cell, a phenomenon mediated by the interaction of an Env with a viral receptor. The budding viral particles “surfed” along the cytonemes and towards the target cell using the actin-myosin cytoskeleton. Filopodia are also involved in the transmission of HIV-1 virions synthesized de novo from productively infected DCs to T cells. Aggarwal et al. found that the accessory protein, Nef, mediates an increase in the number of DC filopodia with immature HIV particles on their tips [106]. Filopodia scan the space to establish contact with the T cell; then, the cells converge, and the DC wraps the T cell to establish a folded VS.

Later, HIV-1 and HTLV-1 cell-to-cell transmission via TNT was reported. TNTs are thin and long intercellular junctions that do not have contact with the matrix proteins, and contain actin, but not tubulin. TNTs are formed between cells of the immune system, neurons, and glial cells, and provide intercellular communication over long distances to transport calcium, genetic material, proteins, and organelles [107]. TNTs between T cells are not open-ended, and are formed by cells coming into contact and then moving apart. TNTs have a diameter of less than 200 nm and a length of about 22 μm, making them 5–10 times longer than filopodia [108]. Env and Gag in HIV-1-infected T cells are colocalized at TNTs, and Gag translocates along the nanotubes, and enters target T cells using the Env/CD4-dependent mechanism. In vivo HIV-1-infected T cells also form TNTs, contributing to viral spread in the lymph nodes of humanized mice [109]. In macrophages, HIV-1 infection increases the number of short (30 μm) and long (150 μm) TNTs; Gag is detected inside the short TNTs, and on the surface of long TNTs [110]. According to a study by Kadiu and Gendelman, viral proteins and RNA can be even found in endosomes within macrophage TNTs [111]. Collectively, these data support the idea that viral particles can be transferred from infected to uninfected macrophages via nanotubes. However, Okafo et al. reported that, although HIV-1-induced nanotubes containing connexin-43 at their tips formed gap junctions with target macrophages, no viral particles were detected at these macrophage–macrophage junctions [112]. Nevertheless, blocking these contacts reduced the level of viral replication and cell-to-cell transmission. The authors suggested that TNTs are not involved in the transfer of viral particles, but rather sensitize target cells to HIV-1 infection. The HIV accessory protein, Nef, has been reported to mediate the formation of TNTs in infected macrophages via the cellular protein M-sec [113], and by enhancing the production of the motor protein myosin-X [114]. Moreover, Nef is capable of translocating from HIV-1-infected macrophages to B cells through nanotubes, where it exerts anti-immune activity by inhibiting the production of antiviral IgG2 and IgA [115].

**Figure 5:**
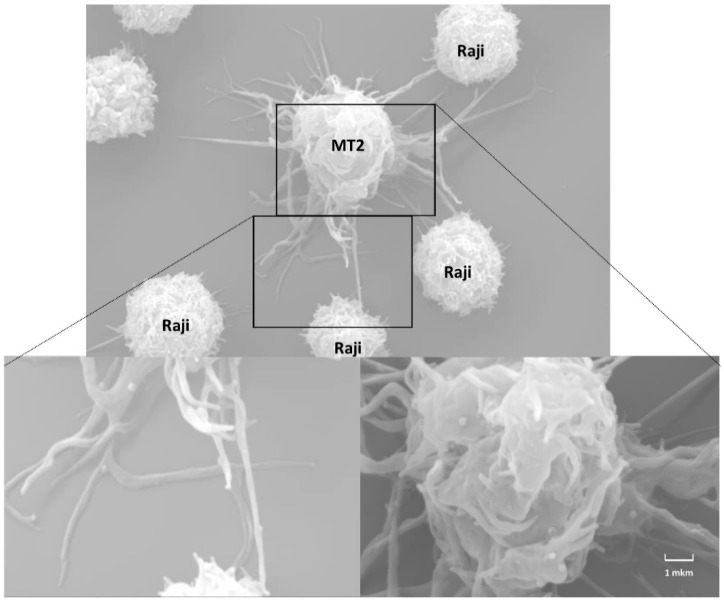
Scanning electron microscopy of HTLV-1 chronically infected cells, MT2, co-cultured with Raji B cells at a 1:5 ratio for 1 h (Mazurov D, unpublished data). Budding virions can be seen on the surfaces of the MT2 cell body and thin protrusions.

The role of nanotubes in the transmission of HTLV-1 was first reported by Van Prooven et al. [116]. The authors called the discovered structures “conduits”, a term that has been applied only to HTLV-1. The viral p8 protein plays an important role in the transmission of HTLV-1 through conduits. p8 is generated by a two-step proteolytic cleavage of the p12 protein encoded by open reading frame 1 (orf-1) in the HTLV-1 genome [117]. p8 increases the number and length of conduits, and enhances intercellular contacts, inducing clustering of LFA-1. Furthermore, p8 is rapidly translocated along conduits into recipient T cells, and suppresses the TCR signaling cascade. This leads to T cell anergy, and attenuates the immune recognition of infected cells. In addition to p8, the viral proteins, Gag and Env, are detected in conduits, whereas the mature viral particles can be at the contacts of a conduit with a cell body, or with another conduit [116]. Gross et al. observed concentration and budding of HTLV-1 at such contacts, and named them “mini VS” [43] (Figure 4d). In our early studies (2003–2004) using scanning electron microscopy, we also observed the formation of conduit-like contacts between a chronically HTLV-1-infected MT2 cell and the surrounding uninfected Raji B cells (Figure 5). The mixture of indicated cells at 1:5 ratio was placed on poly-L-lysine coated plastic surface, and incubated at 37 °C for 1 h. Then, cells were prepared for microscopy. One infected cell could make thin contacts with several nearby uninfected cells. In zoomed-in areas, the budding viral particles could be seen both on the surface of the cell body and along the conduits. However, we also observed the formation of the thin membrane protrusions between Raji cells in the absence of MT2 cells (data not shown), which may reflect the mobility of plastic-seeded cells, which make close contacts to each other, then move away, staying connected via membrane protrusions. Thus, it is unclear whether this cellular model reflects the cell-to-cell transmission of HTLV-1, and how relevant is it to the natural infection of T cells despite some evidence for infection of B cells in vivo.

Based on HIV and HTLV TNT studies, an analogy can be drawn between the p8 protein from HTLV-1, and Nef from HIV-1. Both proteins localize at the IS, suppress TCR function, and induce conduit/TNT formation. They translocate to recipient cells via TNTs, causing cell anergy and viral escape from immune surveillance [118]. The HTLV-1 regulatory protein, Tax, which, in producer cells, plays a central role in the cell-to-cell transmission of HTLV-1 across VS, can also be found in conduits. Tax is not incorporated into viral particles, but affects many signaling pathways, including NF-kB [119], and can also induce changes in recipient cells, sensitizing them to HTLV-1 infection [120].

It should be noted that HIV-1 and HTLV-1 sensitize cells to infection not only through conduits/TNT contacts, but also by using exosomes or extracellular vesicles. Exosomes play an important role in intercellular communication, and contain microRNAs, proteins, mRNAs, enzymes, lipids, and carbohydrates [121]. In in vitro experiments, exosomes secreted by HTLV-1-infected HUT102 cells and containing the viral protein Tax, increased the number of intercellular contacts, which led to enhanced viral transmission. In in vivo experiments, pretreatment of humanized mice with such exosomes increased the infection of different organs and tissues by HTLV-1 [122]. Tax was also found in a soluble form, which can work as virokine, and affect the function of uninfected lymphoid cells [123], astrocytes [124], and neuronal cells [125], implicating its role in HAM/TSP pathogenesis. It was reported that exosomes secreted by HIV-1-infected cells contained a *trans*-activation response element (TAR), an RNA structure involved in the activation of the viral 5′-LTR promoter, as well as cellular RNase III enzymes, Dicer and Drosha. Incubation with such exosomes increased the permissiveness of recipient cells to HIV-1 infection, and inhibited the apoptosis of infected cells [126].

## 5. Relevance of Different Modes of Transmission to Viral Spread In Vivo

Much of the knowledge about different intercellular contacts mediating viral transfer has been obtained in two-dimensional (2D) cell culture settings. This raises the fundamental question of whether all or some of the transmission mechanisms are important for virus dissemination in vivo, where the infected cell and the target cell reside in a highly motile environment. Hence, studies involving 3D cultures, animal models, and patient materials can help understand the importance of each mechanism.

Unlike HTLV-1, HIV can efficiently utilize both cell-free and cell-to-cell modes of transmission for spreading in vitro or in vivo. Indeed, observation of lymphadenopathy and HIV accumulation in the patient’s lymph nodes at the early stages of the disease [127,128] suggest that close lymphocyte contacts may facilitate virus transmission. However, without special experimental settings, it is hard to dissect different modes of HIV transmission in vivo. Studies in humanized mice demonstrate that HIV-infected T cells retain the ability to circulate and migrate to the lymph nodes and spleen, where they can interact with uninfected cells, arrest their motility to establish contact-dependent transfer of HIV, and then move apart from each other with the formation of long tethering membrane protrusions [109,129]. From these studies, it is not clear whether the transmission occurs early at VS, or continues via connecting nanotubes. Muruoka et al. [109] have found that some cells at VS are fused in bone marrow-liver-thymus (BLT) humanized mice. As mentioned above, VS formation includes some mechanisms that prevent cell–cell fusion. The role of syncytial cells in virus spread remains unclear, and is discussed in a recent review [130]. Law et al. have shown that cell-to-cell infection enhances co-transmission of two different strains of HIV-1 in vitro and in vivo [129]. This is well consistent with our results obtained using cell lines and two reporter vectors, inGFPt and in-mCherry [131], suggesting that cell-cell contact can increase multiplicity of infection and diversity of HIV. Nonetheless, the high doses of infected cells used to challenge cells or animals in these experiments are likely irrelevant to the dynamics of viral propagation during acute HIV infection in humans. The tissue-like 3D culture systems greatly complement studies in vivo, and demonstrate that a 3D environment limits infection by cell-free virions, but promotes cell-associated HIV-1 transmission between T lymphocytes [132]. DCs that have captured HIV can migrate in 3D collagen towards lymph-node-homing chemo-attractant gradients, and establish infectious synapse with CD4+ lymphocytes [133], implicating the role of DC-mediated dissemination of HIV upon sexual transmission. Despite some evidence that VS can promote HIV spread in vivo, Galloway et al. showed that HIV cell-to-cell transmission, but not cell-free virions, induces pyroptotic death of target cells, limiting the infectious spread via cell contacts [134].

Usually, the primary infection of humans with HTLV-1 is asymptomatic. Therefore, it is difficult to study the early stages of HTLV-1 replication in humans. It is generally accepted that HTLV-1 is initially transmitted through cell-to-cell contacts followed by proviral DNA increase due to the clonal expansion of infected T lymphocytes. However, Laydon et al. demonstrated that during asymptomatic infection, HTLV-1 can be produced de novo, and increase the clonal diversity of infected lymphocytes [135]. Some mechanisms of HTLV-1 replication and virus-associated disease progression can be learnt from animal models [44,122,136,137]. However, in vivo experiments demonstrating the role of VS during acute infection, as was shown using intravital fluorescence microscopy for HIV (see above) and MLV [138], have not been reported for HTLV-1. The role of biofilms in HTLV-1 infection has been additionally confirmed, using only the primary salivary gland epithelial cells [139].

Thus, much less is known about the mechanisms of HIV and HTLV-1 cell-to-cell transmission in vivo in comparison to different transmission modes described in vitro.

## 6. Conclusions

Viruses can hijack the mechanisms of intercellular communication to enhance their transmission and replication. Though HIV-1 can effectively use both the cell-free and cell-to-cell modes of transmission, HTLV-1 spread relies almost exclusively on cell-to-cell transmission. Multiple forms of cell-cell contacts have now been described, across which the transfer of retroviruses has been observed. However, the type most important for the spread of the virus in vitro and in vivo remains to be elucidated in future studies. The importance of the cell-to-cell transmission mechanism for viral dissemination is evidenced by the resistance of cell contact-mediated infection to neutralizing antibodies, which has been demonstrated for HIV-1 [140] and SARS-CoV-2 [33,141]. The detailed mechanisms of viral cell-to-cell transmission have not been uncovered so far. Understanding these mechanisms is necessary to properly target them, and develop transmission-specific antiviral drugs.

## Data Availability

The data are available from the corresponding author upon a reasonable request.
